# Pleural Effusions and Pericarditis: A Retrospective Cohort Study of Patients Undergoing Cardiac Magnetic Resonance Imaging

**DOI:** 10.7759/cureus.23599

**Published:** 2022-03-29

**Authors:** Raheel Ahmed, Avinash Aujayeb

**Affiliations:** 1 Cardiology Department, Northumbria Healthcare NHS Foundation Trust, Newcastle, GBR; 2 Respiratory Medicine Department, Northumbria Healthcare NHS Foundation Trust, Newcastle, GBR

**Keywords:** exudate, pleural effusion, pericarditis, mri, myocarditis

## Abstract

Background

Pleural effusions can occur due to acute pericarditis and can necessitate intervention. We sought to add to the evidence base by performing a retrospective review of patients presenting to the advanced cardiac imaging unit with pericarditis and pleural effusion to determine laterality, trends in pleural fluid analyses, and the need for pleural intervention. Local ethical (Caldicott) approval was obtained for this study.

Methodology

Descriptive statistical methodology was applied with continuous data presented as mean (standard deviation, SD; range) and categorical variables as frequencies or percentages.

Results

In 60 patients with pericarditis, 24 (39%) had pleural effusions on contemporaneous imaging. The mean age of the study population was 63.3 years (range: 20-83), and 17 patients were males. Diagnoses were viral (five), rheumatological (one), amyloidosis (one), listeria (one), and the rest idiopathic (17). Four effusions were only left-sided, one right-sided, and 20 bilateral. Ten pleural taps were performed, one for a unilateral effusion and nine for one side being bigger than the other. The mean pH was 7.46 (7.33-7.6), mean lactate dehydrogenase was 210 (74-393 U/L), mean fluid protein was 36.1 (19-56 g/L) (four effusions exudative/three transudative), mean glucose was 5.8 (4.8-6.8 mmol/L), and all cytologies were negative. Five patients underwent large volume aspirations for symptom control. Three indwelling pleural catheters (IPC) were placed for treatment refractory effusions. There was one pleural space infection in six months related to an IPC. There were three deaths at 12 months, with none related to pericarditis.

Conclusions

Pleural effusions associated with pericarditis are usually small, bilateral, and exudative. Treatment refractory cases require pleural intervention, with aspirations, drains, and IPCs being viable options. Further prospective studies are warranted.

## Introduction

The pericardium has visceral and parietal layers separated by the pericardial cavity. Normally, between 15 and 50 mL of plasma ultrafiltrate circulates in the cavity. Inflammation of the pericardium, pericarditis, can occur due to a number of different causes (viral/bacterial infections, autoimmune pathology, tuberculosis, diffuse carcinomatosis), although a significant proportion, up to 55% in Western countries, are deemed idiopathic [1].

Pericarditis can present with chest pain and systemic inflammatory symptoms such as fever, fatigue, and malaise. Classic changes on an electrocardiogram (ECG) are widespread ST-elevation (typically concave up) with reciprocal ST-depression in leads aVR and V1. A pericardial rub can be present on auscultation, and pericardial effusion can be present on contemporaneous transthoracic echocardiography (TTE). While TTE is the first-line investigation for pericarditis, cardiac magnetic resonance (CMR) imaging can be a sensitive and specific tool for the evaluation of pericarditis as the inflamed pericardium is bright and thickened on T2-weighted imaging due to edema and enhanced after contrast injection (late gadolinium enhancement) [1,2]. The 2015 European Society of Cardiology (ESC) guidelines puts cardiac CT or CMR as second-line investigations to diagnose pericarditis. However, in real-life clinical practice, CMR is increasingly being used for definitive diagnosis [3].

Northumbria Healthcare NHS Foundation Trust (NHCT) runs a very large pleural service and offers advanced pleural procedures, such as pleural biopsies, indwelling pleural catheters (IPCs), and local anesthetic thoracoscopy [4]. Pleural effusions can occur with pericarditis, and there are several reviews of pleural effusions and pericarditis that have been described, most notably in vasculitides and rheumatological conditions. There are also two case series of patients in pleural effusion databases which were retrospectively analyzed for pericarditis. These are described in greater detail in the later parts of this paper.

Despite the above evidence, no local review has ever been performed. NHCT is also an advanced cardiac imaging center and patients undergoing CMR are rigorously cataloged, and searches within that database are practical and feasible. Thus, we aimed to describe a cohort of patients with CMR-proven pericarditis who had contemporaneous pleural effusions along with their relevant subsequent investigations and outcomes.

## Materials and methods

Local Caldicott approval from NHCT was obtained for a retrospective cohort study of all patients who underwent CMR locally from July 2016 to July 2020 (reference: RPI-1315).

All CMR scans were reviewed to include only patients with CMR-proven pericarditis. The patients were analyzed to determine if they had pleural effusions on contemporaneous imaging (for example, chest radiographs or computed tomography).

Demographics, pleural fluid characteristics, cardiac markers, and outcomes of the patients were collected from the electronic notes.

A descriptive statistical methodology was applied with continuous data presented as median and interquartile ranges (IQRs) and categorical variables as frequencies or percentages. The analysis was performed on Microsoft Excel 2019 (Microsoft 365).

## Results

A total of 4,368 CMRs were performed between July 2016 and July 2020. All reports and imaging were reviewed and those showing pericarditis (60, 1.4%) were analyzed. Of those 60, 24 had contemporaneous pleural effusions and were reviewed for this study.

Diagnoses were viral (five), rheumatological (systemic lupus erythematosus) (one), amyloidosis (one), listeria (one), and the rest were deemed idiopathic (16; see Table 1).

**Table 1:** Number of cases of pericarditis by etiology.

Etiology of pericarditis	Number of cases
Viral infections	5
Rheumatological	1
Amyloidosis	1
Listeria infection	1
Idiopathic	16

There were three left-sided effusions, one right-sided effusion, and the rest (21) were bilateral. Small effusions were defined as the appearance of a meniscus of increased density at the costophrenic angle. Small effusions were defined as those occupying less than one-third of the hemithorax, large effusions as more than two-thirds of the hemithorax, and moderate ones as more than one-third and less than two-thirds of the hemithorax. There were 11 small effusions, 12 moderate effusions, and one large effusion (see Table 2).

**Table 2:** Laterality and size of effusions.

Effusion characteristics	Number of cases
Left-sided only	3
Right-sided only	1
Bilateral	21
Small effusions	11
Moderate effusions	12
Large effusions	1

Nine pleural taps (aspiration of pleural fluid for diagnosis only, 50 mL maximum), one for a moderate unilateral effusion, and all others for bilateral effusions, with one effusion being bigger than the other. Median pleural fluid pH was 7.49 (IQR = 0.24, no data in two patients), median pleural lactate dehydrogenase (LDH) was 246 U/L (IQR = 162.5), median plasma LDH was 250 U/L (IQR = 180, no data in four), median fluid protein was 38 g/L (IQR = 16.7, no data in one), median plasma protein was 66 g/L (IQR = 9.5, no missing data), and median fluid glucose was 5.9 mmol/L (IQR = 1.4, no data in two). All samples were exudative in nature, according to Lights criteria, which is well established [5]. All pleural fluid cytology was negative for malignancy, and none underwent further pleural biopsies through medical thoracoscopy, for example.

Six therapeutic aspirations were done for symptom control (breathlessness), and one of those had a small-bore intercostal drain. All patients were treated with a combination of ibuprofen, colchicine, and steroids for at least three weeks. Various other treatments were administered (diuretics, beta-blockers, antibiotics), and four required cardiothoracic input (offered regionally at a tertiary center) for drainage of large pericardial effusions and pericardial stripping. Due to re-accumulation of the pleural effusions despite treatment, three IPCs were inserted, with resultant pleurodesis occurring in two patients. One patient developed an IPC-related empyema which required intrapleural fibrinolysis and then eventual removal of the IPC. The pleural fluid did not recur. Description of pleural fluid analysis and pleural procedures done are presented in Table 3.

**Table 3:** Description of pleural fluid analysis and pleural procedures done. IQR: interquartile range

Pleural fluid analysis and procedures	Number of cases
Pleural taps	9
Therapeutic aspirations	6
Intercostal drain	1
Indwelling pleural catheters	3
Median pleural fluid pH	7.49 (IQR = 0.24)
Median pleural lactate dehydrogenase	246 U/L (IQR = 162.5)
Median fluid protein	38 g/L (IQR = 16.7)

For those with bilateral pleural effusions, there was no evidence of common causes of bilateral pleural effusions (hypoalbuminemia [liver dysfunction or critical care illness], renal failure, overt heart failure) in 15 patients. Six N-terminal pro-B-type natriuretic peptide levels were measured, all in patients with bilateral pleural effusions. Only three of the patients had elevated levels consistent with heart failure (>400 ng/L). Mortality at six and 12 months was 8% (two patients: one due to heart failure, one unknown cause of death).

## Discussion

A PubMed search with the words (pleuritis) OR (pleural effusion) AND (pericarditis) from inception to December 2021 revealed 1,346 articles. There was only one randomized controlled trial looking at colchicine and preventing postoperative pleural effusions [6], which was not relevant to this article. The most relevant articles, including case-based reviews and systematic reviews, are described below.

First, there were two case series that were derived from databases of patients with pleural effusions and retrospectively analyzed for pericarditis. Lazaros et al. analyzed 177 consecutive patients with acute pericarditis over a median follow-up period of 12 months [7]. The incidence of pleural effusions was 53.1% (n = 94) with just over 50% being bilateral. Their presence was strongly associated with C-reactive protein levels at admission, age, and female gender. Bilateral effusions were also associated with risk of cardiac tamponade. However, there was no analysis of the pleural fluid. Porcel et al. showed that 4% of 3,077 pleural effusions were due to pericardial disease, with the caveat that bilateral pleural effusions are often present in patients with pericarditis and are too small to aspirate and evaluate [8]. However, given that pleural fluid analysis is almost never reported, the same authors sought to evaluate the imaging and pleural fluid of effusions associated with acute idiopathic pericarditis and/or post-cardiac injury syndrome. In the largest and most recent study [9], 82 patients with acute idiopathic pericarditis were analyzed; just under half were males and the median age was 67 years. Bilateral pleural effusions were present in 41% and left-sided in 55%. Overall, 99% of effusions were exudative, but of note, pericardial imaging was not available in approximately 40% of patients.

Pleural effusions are thought to develop in the context of pericarditis due to contiguous inflammation (pleura and pericardium are in direct contact at the mediastinum) or the same process affects the pericardium and the pleura (for example, cancer dissemination) [1,2,8,9]. Thus, systemic inflammation is at play, as evidenced by Thompson et al. [10], who found that 88 of 1,058 patients (8.3%) with granulomatosis and polyangiitis, 27 of 267 (10.1%) with microscopic polyangiitis, and 35 of 201 (17.4%) with eosinophilic granulomatosis with polyangiitis had pleural and pericardial involvement. This has also been described in patients with rheumatoid arthritis, with one notably requiring intravenous immunosuppression, conventional chest drainage, and surgical treatment for the pleural effusions [11]. Other causes described in the literature include chlamydia pneumonia [12], yellow nail syndrome [13], and following anti-cancer treatment [14]. This list is not exhaustive, and a full review is beyond the scope of this article.

Our study has the strength of having contemporaneous cardiac imaging (CMR) which is a very sensitive and specific tool for detection of pericardial inflammation. Because our center does not have a cardiac surgery department, we do not see any of the post-cardiac surgery pericarditis and pleural effusion. We describe similar findings that effusions are more likely to be small and bilateral, and this real-life analysis shows that they are unlikely to be intervened upon. Moreover, treatment of pericarditis helps with pleural clearance but recurrence can occur and requires further intervention.

This being a real-life retrospective study, it has significant limitations. A large number of data points such as C-reactive protein and brain natriuretic peptide levels were incomplete as any tests performed would have been at the discretion of the physician. Pleural fluid indices were also incomplete and at the physician’s discretion. We also did not analyze all ECGs performed in the trust, which might have also picked up a number of pericarditis cases, and those patients might not need a CMR. Further, because the above data is from a very select group of patients, thus significant bias is present.

However, in our view, we achieved what we set out to analyze, that is, pleural effusions in those with pericarditis for several patients. Our study sample, while small, is probably representative of the wider population. Because of the observational nature of our study, we will refrain from stating conclusions with utmost confidence: the trends we observed are that effusions in pericarditis tend to be mostly bilateral, patients are mostly male, effusions are not transudative (probably signifying inflammation), and pleural interventions are reserved for those not responding to treatment.

There is a growing body of evidence regarding the role of IPCs in managing non-malignant effusions. The recently published REDUCE study showed that IPCs did not control breathlessness any better than repeated thoracenteses but they reduced the number of pleural procedures patients required and might improve the quality of life [15]; 1/31 IPCs (3%) were infected [15]. We have previously reported our local IPC experience in so-called benign effusions and found that our infection rates are much higher than the quoted literature and when compared to our overall cohort [16,17]. Our numbers are too small to provide any discrete conclusions but can stimulate discussion around this topic.

## Conclusions

We have shown that pleural effusions associated with pericarditis are usually small, bilateral, and exudative. Treatment refractory cases require pleural intervention, with aspirations, drains, and IPCs being viable options. It is very clear that further research is required, namely, a multicenter prospective study examining all patients with idiopathic pleuritis, describing the epidemiology, and, if feasible, sampling their pleural effusions for detailed analysis and correlating with cardiac and inflammatory markers as well as contemporaneous imaging.
